# Supplementary material for: MetaRibo-Seq measures translation in microbiomes
Source: Nat Commun. 2020 Jun 29;11:3268. doi: 10.1038/s41467-020-17081-z (PMC7324362; doi:10.1038/s41467-020-17081-z)
Supplement: Supplementary file 10 — Supplementary Data 7 [file 41467_2020_17081_MOESM10_ESM.zip › File2/Confidence_VeryHigh_Taxonomy/112649_out.krona.html]

Javascript must be enabled to view this page.

members
magnitude
magnitudeUnassigned
count
unassigned
taxon
rank

112649\_out

9

9
superkingdom
2

1239
phylum
9

9
91061
class

186826
order
9

9
1300
family

9
1301
genus

1305

SRS015985\_contig\_number\_144SRS015989\_contig\_number\_159SRS016746\_contig\_number\_37436SRS017511\_contig\_number\_93SRS043018\_contig\_number\_38003SRS076926\_contig\_number\_15148SRS143616\_contig\_number\_8923SRS146750\_contig\_number\_1009SRS149585\_contig\_number\_1690
species
9
